# Factors Influencing Women's Decision to Participate or Not in a Surgical Randomised Controlled Trial for Surgical Treatment of Female Stress Urinary Incontinence

**DOI:** 10.1155/2013/139813

**Published:** 2013-09-17

**Authors:** Alyaa Mostafa, James N'Dow, Mohamed Abdel-Fattah

**Affiliations:** ^1^Division of Applied Health Sciences, University of Aberdeen, 2nd Floor, Aberdeen Maternity Hospital, Foresterhill, Aberdeen AB25 2ZD, UK; ^2^Division of Applied Health Sciences, University of Aberdeen, Academic Urology Unit, 2nd Floor Health Sciences Building, Foresterhill, Aberdeen AB25 2ZD, UK

## Abstract

The study aims to explore the potentially influential factors affecting women's decision to accept/decline participation in surgical randomised trial using a novel acceptance/refusal questionnaire (ARQ). All women who were eligible to participate in SIMS-RCT were asked to complete the relevant section (acceptance/refusal) of the ARQ. Women reported its degree of relevance for their decision on a six-point Likert scale (0 = highly irrelevant, 5 = highly relevant). 135 (98%) and 31 (70%) women completed the acceptance and refusal sections of the ARQ, respectively. The most influencing factor in women's acceptance was the anticipation of “potential personal benefit”; percentage of relevance (POR) was 91.9%, followed by interest in helping others by “supporting innovative medical research”; POR was 87.7%. Most influencing factor in refusal for participation was “do not have time for follow-up”; POR was 56.8%, followed by “do not like the concept of randomisation”; POR was 54.4%. In conclusion, this study identifies the most influential factors relevant to women decision-making whether or not to participate in RCTs assessing surgical interventions for female stress urinary incontinence (SUI). A number of factors leading to refusal of participation are potentially correctable leading to better recruitment rates in future RCTs.

## 1. Introduction

Randomised controlled trials (RCTs) are considered the gold standard for the evaluation of the effectiveness and safety of healthcare interventions and are widely accepted as the most powerful research method in evaluating health technologies, principally because they minimise selection bias [1]. RCTs depend entirely on volunteering participants, and therefore one of the main challenges facing RCTs is adequate recruitment [2, 3]. Unfortunately, large number of clinical trials fail to achieve their recruitment target [4, 5], leading to poor statistical power and often inconclusive results [6, 7]. A survey [8] of 41 multicentre RCTs by the National Institute of Health in North America reported that 14/41 trials (34%) recruited less than 75% of their recruitment goal. Similarly, out of 114 RCTs in the United Kingdom (UK) between 1994 and 2002, only 31% of trials achieved their anticipated recruitment target within the initial recruitment projection time frame, and 53% required an extension for recruitment period [9]. Extension of the initially planned recruitment period can lead to recruitment fatigue and will have significant impact on trial funding and on the work load of the whole trial team [10].

A number of studies have identified number of barriers for participating in clinical nonsurgical RCTs such as patients' strong preference for or against certain treatment [11, 12], trial demands, and time constraints such as, additional time/expenses required for further follow-up commitments [13–15].

Surgical randomised trials fundamentally differ due to the invasiveness of the interventions, the patients' anxiety on potential surgical morbidity, and/or lack of efficacy data that will inevitably characterise the study arm/new intervention. In addition, the motivational differences of patients allocated to the study arm/new intervention versus the standard treatment may have an important influence in participants' retention within surgical RCTs particularly when blinding patients is not possible [16]. Some patients accept postoperative pain and certain postoperative complication as “part and parcel” of the surgical package, while their main emphasis would be on the effectiveness of the procedure; others will have minimal tolerance to postoperative complications especially with minimal invasive procedures. Hence, motivational differences can be considered as one of the driving leads for patient's choice to participate or not in surgical RCTs.

There is a paucity of data in the current literature on the potential patient's barriers/incentives for participation in surgical RCTs; the concept of randomised allocation for treatment [17], desire, or willingness to help others [18], potential for individual benefit [19, 20], and the understanding of trial details and randomisation process [17, 21], issues related to the surgery such as indication of surgery, type of interventions, and surgeons experience [14, 22–24] were potentially influential in patients' decision to participate in surgical RCTs. Interestingly, the little existing evidence is mostly derived from qualitative studies, and therefore quite small cohorts were assessed. 

In this study, we aim to explore the influential factors affecting women's decision on recruitment and participation in a multicentre surgical RCT comparing a new surgical intervention versus the standard surgical treatment of female stress urinary incontinence (SUI).

## 2. Materials and Methods

The SIMS-Trial is a multicentre RCT comparing a relatively new procedure “Single-incision mini slings (SIMS-Ajust)” versus a standard midurethral sling (SMUS-TVT-O), in management of female SUI [25]. Women were recruited in 6 UK centres, in the period between October 2009 and October 2010, and the 1-year outcomes have been published [26]. 

During the pretrial counselling, women were informed that the existing evidence for SIMS was quite limited and showed better tolerability to be done under local anaesthesia (LA) and lower incidence of post-operative pain, shorter hospital stay, quicker recovery, and earlier resumption of day-to-day activities and return to work [27]. However, women were fully informed of the lack of long-term outcome data for the SIMS and that SMUS in the control arm (TVT-O) was considered as the standard treatment and is currently the most common primary continence procedure worldwide [28]. Women were also informed that TVT-O is exclusively performed under general anaesthesia (GA) anaesthetic [29] and has shown to have objective and patient-reported cure rates of 73–82% and 70–74% with up to 5 years or followup [30]; however, can be associated with a relatively high incidence of short-term postoperative groin/thigh pain [31].

The protocol for SIMS-RCT was published on the public domain (www.clinicaltrials.gov) in October 2009 and was approved by the North of Scotland Research Ethics Committee who approved the administration of a purpose-designed “acceptance/refusal questionnaire (ARQ)” to all eligible women. In summary, women were included if they had SUI and had failed or declined pelvic floor muscle training (PFMT), which is the first line of conservative management of SUI prior to the surgical intervention [32]. Women with pelvic organ prolapse (≥POP-Q Stage 2), previous continence surgery, concomitant surgery, or neurological conditions were excluded. The study was adequately powered (95% power) to detect a clinically significant 25% treatment effect (1-point difference on visual analogue scale (VAS), and used a computer-generated randomisation with adequate allocation concealment. All eligible women were invited to participate and received a preoperative package including patient information sheet with details about the type of procedures and study details. In addition, the preoperative pack included symptom severity and quality of life (QoL) assessment questionnaires and the ARQ. 

This study for evaluating the factors influencing women's decision to participate or not in surgical RCTs was embedded in the SIMS-RCT. Women were informed that completing the ARQ was completely voluntary and that their responses will be handled confidentially and anonymous and will not affect their further management or care. Participants completed the ARQ in their own privacy either during their hospital clinic or at their home when they received their preoperative packs. Women were able to clarify their understanding of any part of the questionnaire with their consultant or the research team.

### 2.1. Acceptance/Refusal Questionnaire (ARQ)

The ARQ was developed based on the best available literature; however, most of the literature is derived from nonsurgical RCTs [19, 33, 34]. Consumer's representatives, patient support group in the Scottish Pelvic Floor Network, were involved in the design of this questionnaire from its early stages; this included a number of modifications to ensure a layman language. Moreover, advice from urogynecology consultants and academics with experience in surgical RCTs was thought (see the appendix).

The ARQ has 2 sections, acceptance and refusal. We have grouped the concepts into 5 key concept groups relating to: (a) views about the trial process and procedures, (b) the possible self-benefits, (c) possible benefit/help to others (known as altruism) (d) personal circumstances during the trial period, and (e) views regarding the interventions compared in the trial. Similar questions were asked in different ways within the questionnaire to assess consistency of responses. For each factor, women were asked to rank its degree of relevance for their decision-making whether to participate or not, on a 6-point Likert scale (ranging from 0 = highly irrelevant to 5 = highly relevant). To determine the content validity of the questionnaire, it ended with an open-ended question for patients to add any other possible influential factors that were not included in the closed items of the questionnaire. The ARQ was then piloted among the first 30 eligible women and was answered by 27; the results showed minimal missing data and no incidents of incomprehensible questions within the acceptance or refusal sections. Furthermore, no other relevant influencing factors were identified from the open-ended question.

A scoring system was developed for the ARQ. The total score for each factor was calculated as the number of respondents for each Likert value (*n*) multiplied by the particular likert value (*L*); that is, *L* = 0,1, 2,…5, such that the final total score for each factor = *n*∗0 + *n*∗1 + *n*∗2 + *n*∗3 +  *n*∗4 + *n*∗5. For both acceptance and refusal, the minimum score is zero. However, the maximum score varies according to the number of respondents (*n*); for the acceptance section of the ARQ, there were 135 respondents, providing a potential total score maximum of 675 (135∗5). In the case of refusal, there were 31 respondents, giving a maximum possible total score of 155 (31∗5). To allow comparison across factors and in-between studies, we calculated the percentage of relevance (POR) for each factor = actual total score for each factor/divided by the potential maximum score, for example, the POR for “the potential personal benefit” = total score/maximum score = 620/675 that is 91.8%.

Data collected were observational, and descriptive analysis was undertaken and presented as percentages for each concept group. Statistical analysis was performed under statistician supervision, using SPSS 18.0 (Chicago, Il, USA).

## 3. Results

**Figure 1:**
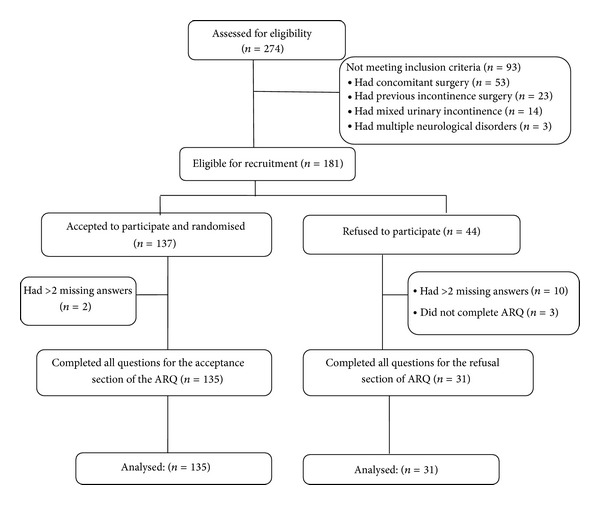
SIMS-Ajust study and ARQ; flow chart of recruited patients.

In the SIMS RCT, 274 patients were referred for surgical management of SUI in all participating centres, and 181 patients were eligible to participate in this trial. 137 patients agreed to participate in the RCT compared to 44 patients who refused to participate. The responses rate was excellent; 135/137 (98.5%) patients completed the acceptance section of the ARQ, and 2/137 (1.5%) patients had >2 missing answers (Figure 1). 31/44 (70%) patients completed the refusal section of the ARQ, 8/44 (18.6%) had >2 missing answers, and 5/44 (11.4%) patients refused to complete the questionnaire without reporting any reasons. 

Baseline characteristics were comparable for women who have completed either section of the ARQ (Table 1).

**Figure 2:**
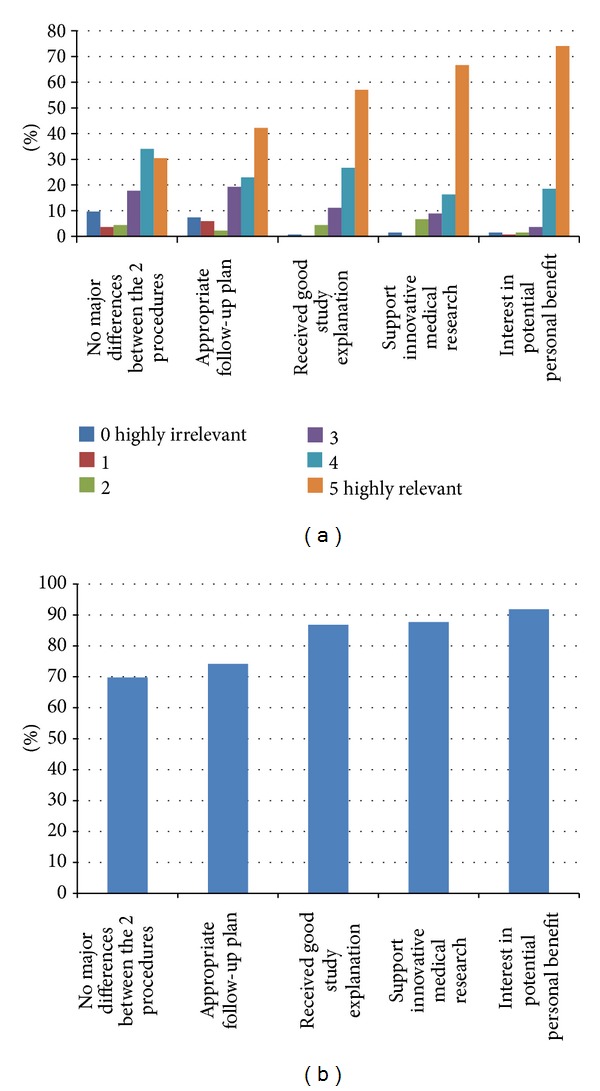
(a) Reasons for accepting to participate, (b) percentage of relevance (POR) for accepting reasons.

135/137 (98.5%) of participating women fully completed the acceptance section in the ARQ. 74% (*n* = 100) rated “potential personal benefit” as highly influential (i.e., rank 5 on the likert scale) in their decision to participate. This was followed by “possible benefit to others (known as altruism)” and “willingness to support innovative medical research” with 67% giving it top rank (5 on likert scale) (Figure 2(a)).  Figure 2(b) summarises the comparison of all factors affecting women's decision to participate in this RCT by the percentage of relevance (POR). Highest POR was for the “potential personal benefit” with a total score of 620/675 (91.8%), followed by “possible benefit to others (known as altruism)” with a total score of 592/675 (87.7%).

**Figure 3:**
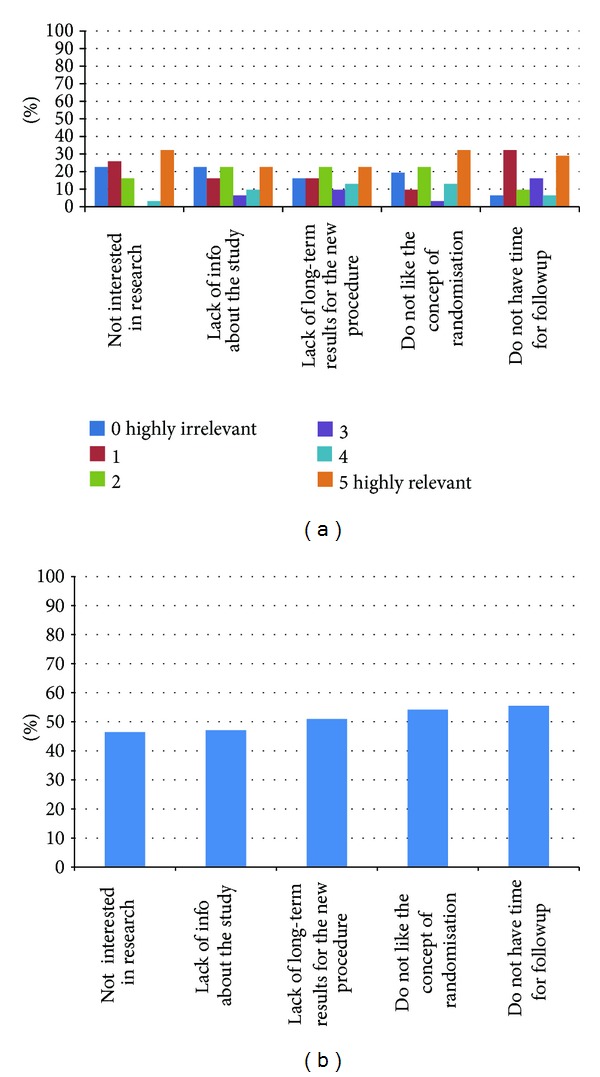
(a) Reasons for accepting to participate, (b) percentage of relevance (POR) for refusal reasons.

Potential factors listed in the refusal section of the ARQ were all closely relevant to women's decision to decline participation in this RCT. 32.5% women ranked “not interested in research” and “do not like the concept of randomisation” as the highly relevant (rank 5 on the likert scale). 29% of nonparticipating women reported that they “do not have time for follow-up process” or are generally “not interested in research,” as the most influential factors in their decision-making (Figure 3(a)).  Figure 3(b) summarises the comparison of all factors affecting women's decision to decline participation in this RCT by the percentage of relevance (POR).

Six women completed the open-ended question at the end of the ARQ: 2 participants and 4 in the declining group. Women added number of reasons that affected their decision-making: “I would like to improve the level of provided healthcare for future patients”; “I am really interested to go to work as quickly as the post-operative recovery will allow.” One woman in the declining group added that she is “so busy and have many family and social issues, so cannot commit to any further follow-up”; further 3 women expressed guilt about not being able to help with research but cannot overcome their preference to have the standard procedure (TVT-O).

## 4. Discussion

Surgical RCTs are expensive to set-up and run, usually requiring significant resources, manpower, and financial investment and therefore every step should be taken to optimise their success. There is a general understanding in the community that patients' recruitment is an integral factor for the success of medical research, yet in practice, fewer patients volunteer to participate in surgical RCTs [35]. 

SIMS RCT is a comparison between two surgical interventions in the management of women with SUI. It is a typical surgical RCT where there is uncertainty regarding the long-term surgical outcome in the study group while there are potential short-term personal benefits such as feasibility to be done under LA, lower postoperative pain and earlier return to normal activities and work. Hence, the SIMS-RCT represented an ideal opportunity to explore the influential reasons in women's decision-making whether or not to participate in surgical RCTs. 

 The recruitment rate was reasonable at 75.7% of eligible women and is comparable with other RCTs in this field. All women were well informed that the completion of the ARQ is entirely voluntary; the response rate for the acceptance group was high (98.5%), while it was 70% for the refusal group. The most influential reasons in women's decision to participate were their interest in potential personal benefits within the new intervention and altruism. These findings are in agreement with previous studies exploring the potential influencing factors for participation in clinical nonsurgical RCTs [18–20].

 Altruism was previously rated as the main motivation for participation in clinical RCTs [18, 36]; however, recent evidence has been more cautious about the role of altruism. It has been suggested that patients can be initially motivated towards participating in a trial based on their willingness to help others or to help research in general; however, this would be heavily influenced by whether there is a potential personal benefit in participating in that particular RCT; this is known as “conditional altruism” [37]. There is no evidence in the literature to compare the percentage of personal benefits and helping others as a motivation to participate in a clinical trial. 

“Good explanation of the study aim, design, and the followup plan” was highly ranked by women in our study as an influential factor in their acceptance decision. This is really a common sense finding and previous literature showed that people's decisions about whether or not to take part in trials depend on their level of understanding of the study aims and steps. Bevan et al. and Bergmann et al. [38, 39] have shown that the lack of study information, especially the clarity of the aim and methods, is a well recognised reason for a patients decision to decline participation in nonsurgical RCTs. Other studies have shown that RCTs with perceived additional demands for inclusion or followup can suffer from difficulties in recruitment and retention [14, 33].

Patient preference to a particular surgery has been previously cited as one of the main barriers for recruitment and postrandomisation retention in surgical RCTs [40]. Plaiser et al. and McCulloch et al. [14, 41] reported significant number of participants withdrawing after randomisation if they receive the more invasive procedure. In our study, the surgical difference between both study arms was perceived by women to be minimal hence was not shown to significantly influence women's decision-making.

There are several factors for eligible women refusing to participate in RCTs. In our study, the most influential factor in women's decision to decline participation was their concerns regarding additional follow-up demands and its associated inconvenience, expenses, and time. Ross et al. [33] have previously shown similar results where additional trial demands and time constraints were seen as barriers for recruitment in nonsurgical RCTs. A number of studies [13, 14] reported patient's concerns regarding potential discomfort within the follow-up process due to potential repeated examination as a significant barrier for participating in RCTs. This is a potentially correctable factor and was not a concern in our RCT as we emphasised on patient-reported outcomes and avoided any postoperative invasive tests or internal examinations that will not be required in routine clinical practice.

The concept of “random” allocation for treatment and receiving treatment on the bases of chance was a highly relevant reason in women's decision to decline participation in our study; similar findings were previously reported in non-surgical RCTs [17]. This highlights that the rational for randomisation and the understanding of the process involved appears to be poorly understood by many participants [21]. Once more, this is a correctable factor and requires more input in the patient information sheet to provide more explanation to the patient and researchers dedicating more time and explanation in the pretrial counselling. 

Previous studies have shown that the lack of efficacy data on the treatment in the study arm and the “fear of the unknown” associated with new interventions are potential factors for refusing to participate in clinical trials [14, 42]. Similarly, we found the lack of long-term efficacy data on SIMS-Ajust (study arm) was one of the influential reasons in women's decision to decline participation.

One of the study strength is presenting the new assessment tool “ARQ” which proved to be useful in assessing the potentially influential factors in the women's decision-making process. The ARQ was structured to target RCTs involving surgical interventions for improvement of QoL rather than life-threatening or cancerous conditions. As the women completing the ARQ were recruited from several centres within Scotland (both urban and rural), it is fair to generalise the results for the wider population. The questionnaire was completed in women's privacy, and therefore their responses were not influenced by external factors and specifically the doctor-patient relationship. The ARQ can be analysed as POR for each factor to allow comparison across factors and in-between studies, despite variation in the number of participants. A main strength of the ARQ is the public engagement in the design of the questionnaire alongside the literature evidence and expert advice. In summary, the ARQ findings represent a good base for further research—into the validation of this promising tool. A potential limitation in our study is the relatively small number of women completing the refusal section of the ARQ as only 25% of eligible women declined participation in the SIMS RCT. 

### 4.1. Study Implications

Better understanding of the influential factors in potential participants' decision-making is crucial if we are to improve our recruitment and retention rates in surgical RCTs. Our study has identified a number of relevant factors which can be modified or improved to enhance recruitment in surgical RCTs in the field of female UI. Relatively simple and cheap measures such as providing complete and thorough information on the RCTs' aims and methods are needed. This needs to be done using easily understandable language, possibly using coloured illustrations, figures and possibly the use of add-back audiovisual technology/web-links. Better patient understanding of the randomisation process and its significance to the robustness of the results is crucial if we are to stand a chance in improving recruitment and retention. Lessons learned from this study will be important in designing large definitive interventional RCTs in the field female UI. 

## 5. Conclusion

The decision to participate or not to participate in surgical trials is likely to be multifactorial and would be better understood by a balance between altruism and considerations for potential personal benefit known as “conditional altruism.” Additional demands on the patients' time, lack of understanding the importance of the randomisation process and clarity of the study details are the most influential, and potentially correctable factors in women's decision to decline participating in surgical RCT. The results of this study provide urogynaecology and surgical researchers with better understanding of the patient's decision-making process when considering participation in RCTs. This should allow them to improve study designs/protocols/patient information/follow-up commitments to allow maximum recruitment.

## Figures and Tables

**Table 1:** Baseline characteristics.

	Acceptance pt. *n* = 135	Refused pt. *n* = 31	*P* value
Age: *n* (%)			
≤25	0 (0%)	0 (0%)	0.342
26–44	46 (34.1%)	16 (51.6 %)	
45–64	73 (54.1%)	12 (38.7 %)	
≥65	16 (11.8%)	3 (9.7 %)	
Parity: *n* (%)			
0	6 (4.4%)	5 (16.1%)	0.120
1	25 (18.5%)	3 (9.7%)	
2	63 (46.7%)	16 (51.6%)	
3	28 (20.7%)	4 (12.9%)	
≥4	13 (9.6%)	3 (9.7%)	
Work status: *n* (%)			
Working	96 (71.1%)	26 (83.8%)	0.405
Not working	28 (20.7%)	3 (9.7%)	
Retired	8 (5.9%)	2 (6.5%)	
Missing	3 (2.2%)	0 (0%)	

## Appendix

### Acceptance/Refusal Questionnaire (ARQ)

*Dear Patient*

If you have chosen to participate in the study, please complete Section A and sign the consent form if not already done. Otherwise please complete Section B.

*Section A.* Please look at the reasons below and grade the degree of relevance for each factor to your decision to participate in this RCT (“zero” is not relevant and “5” is highly relevant”)

- I see no major differences between the 2 procedures:
-  0 1 2 3 4 5
- The follow-up plan is appropriate and convenient:
-  0 1 2 3 4 5
- I received good study explanation:
-  0 1 2 3 4 5
- I would like to support innovative medical research:
-  0 1 2 3 4 5
- I am interested in a potential personal benefit:
-  0 1 2 3 4 5
- Other reasons: Please expand: ………………

*Section B.* If you have chosen not to participate in the study it would be very helpful if you could let us know the most important reason(s) for your decision by completing Section B. However you don't have to state any reasons and you should be sure that it won't affect your medical care.

*I have declined to participate or withdrawn from this study because* (Please look at the reasons below and grade their relevance to you where “zero” is not relevant and “5” is highly relevant”)

- I am not interested in research:
-    0 1 2 3 4 5
- I am not well informed about study:
-    0 1 2 3 4 5
- There is a lack of long-term results for the new procedure:
-    0 1 2 3 4 5
- I do not like the concept of randomisation in this study:
-    0 1 2 3 4 5
- I do not have time for the follow-up plan:
-    0 1 2 3 4 5
- Other reasons: Please expand: ………………
